# Patients with COVID-19: in the dark-NETs of neutrophils

**DOI:** 10.1038/s41418-021-00805-z

**Published:** 2021-05-24

**Authors:** Maximilian Ackermann, Hans-Joachim Anders, Rostyslav Bilyy, Gary L. Bowlin, Christoph Daniel, Rebecca De Lorenzo, Mikala Egeblad, Timo Henneck, Andrés Hidalgo, Markus Hoffmann, Bettina Hohberger, Yogendra Kanthi, Mariana J. Kaplan, Jason S. Knight, Jasmin Knopf, Elzbieta Kolaczkowska, Paul Kubes, Moritz Leppkes, Aparna Mahajan, Angelo A. Manfredi, Christian Maueröder, Norma Maugeri, Ioannis Mitroulis, Luis E. Muñoz, Teluguakula Narasaraju, Elisabeth Naschberger, Indira Neeli, Lai Guan Ng, Marko Z. Radic, Konstantinos Ritis, Patrizia Rovere-Querini, Mirco Schapher, Christine Schauer, Hans-Uwe Simon, Jeeshan Singh, Panagiotis Skendros, Konstantin Stark, Michael Stürzl, Johan van der Vlag, Peter Vandenabeele, Ljubomir Vitkov, Maren von Köckritz-Blickwede, Cansu Yanginlar, Shida Yousefi, Alexander Zarbock, Georg Schett, Martin Herrmann

**Affiliations:** 1 Institute of Pathology and Molecular Pathology, Helios University Clinic Wuppertal, University of Witten/Herdecke, Wuppertal, Germany; 2 Institute of Functional and Clinical Anatomy, University Medical Center of the Johannes Gutenberg-University Mainz, Mainz, Germany; 3 Medizinische Klinik und Poliklinik IV, Klinikum der Universität München, Ludwig-Maximilians University Munich, Munich, Germany; 4 Danylo Halytsky Lviv National Medical University, Lviv, Ukraine; 5 Department of Biomedical Engineering, University of Memphis, Memphis, TN USA; 6 Department of Nephropathology, Friedrich-Alexander University (FAU) Erlangen-Nürnberg, Erlangen, Germany; 7 Università Vita-Salute San Raffaele & IRCCS San Raffaele Scientific Institute, Milano, Italy; 8 Cold Spring Harbor Laboratory Cancer Center, Cold Spring Harbor, NY USA; 9 Institute for Biochemistry and Research Center for Emerging Infections and Zonoses, University of Veterinary Medicine Hannover, Hannover, Germany; 10 Area of Cell and Developmental Biology, Centro Nacional de Investigaciones Cardiovasculares, Madrid, Spain; 11 Department of Internal Medicine 3 - Rheumatology and Immunology, Friedrich-Alexander-University Erlangen-Nürnberg (FAU) and Universitätsklinikum Erlangen, Erlangen, Germany; 12 Deutsches Zentrum für Immuntherapie (DZI), Friedrich-Alexander-University Erlangen-Nürnberg and Universitätsklinikum Erlangen, Erlangen, Germany; 13 Department of Ophthalmology, University of Erlangen-Nürnberg, Erlangen, Germany; 14 Division of Intramural Research, National Heart, Lung and Blood Institute, Bethesda, MD USA; 15 Division of Cardiovascular Medicine, University of Michigan, Ann Arbor, MI USA; 16 Systemic Autoimmunity Branch, National Institute of Arthritis and Musculoskeletal and Skin Diseases, National Institutes of Health, Bethesda, MD USA; 17 Division of Rheumatology, University of Michigan, Ann Arbor, MI USA; 18 Department of Experimental Hematology, Institute of Zoology and Biomedical Research, Jagiellonian University, Krakow, Poland; 19 Snyder Institute for Chronic Disease, University of Calgary, Alberta, Canada; 20 Department of Internal Medicine 1, Friedrich Alexander University Erlangen-Nuremberg and Universitätsklinikum Erlangen, Erlangen, Germany; 21 Cell Clearance in Health and Disease Lab, VIB-UGent Center for Inflammation Research (IRC), Ghent, Belgium; 22 Department of Biomedical Molecular Biology (DBMB), Ghent University, Ghent, Belgium; 23 First Department of Internal Medicine, Department of Medicine, University Hospital of Alexandroupolis, and Laboratory of Molecular Hematology, Democritus University of Thrace, Alexandroupolis, Greece; 24 School of Health Care Administration, Oklahoma State University Center for Health Sciences, Tulsa, OK USA; 25 Division of Molecular and Experimental Surgery, Translational Research Center, Department of Surgery, University Medical Center Erlangen, Friedrich-Alexander University Erlangen-Nürnberg, Erlangen, Germany; 26 Department of Microbiology, Immunology and Biochemistry, University of Tennessee Health Science Center, Memphis, TN USA; 27 Singapore Immunology Network (SIgN), A*STAR, Biopolis, Singapore, Singapore; 28 Department of Otolaryngology, Friedrich-Alexander-University Erlangen-Nürnberg, Head and Neck Surgery, Universitätsklinikum Erlangen, Erlangen, Germany; 29 Institute of Pharmacology, University of Bern, Inselspital, INO-F, Bern, Switzerland; 30 Medizinische Klinik I, Klinikum der Ludwig-Maximilians-Universität, Munich, Germany; 31 Department of Nephrology, Radboud Institute for Molecular Life Sciences, Radboud University Medical Center, Nijmegen, The Netherlands; 32 Cell Death and Inflammation Unit, VIB-UGent Center for Inflammation Research (IRC), Ghent, Belgium; 33 Department of Biomedical Molecular Biology (DBMB) and Methusalem Program, Ghent University, Ghent, Belgium; 34 Department of Biosciences, Vascular and Exercise Biology Unit, University of Salzburg, Salzburg, Austria; 35 Clinic of Operative Dentistry, Periodontology and Preventive Dentistry, Saarland University, Homburg, Germany; 36 Department of Anesthesiology, Intensive Care and Pain Medicine, University Hospital Münster, Münster, Germany

**Keywords:** Immunological disorders, Infectious diseases, Respiratory tract diseases

## Abstract

SARS-CoV-2 infection poses a major threat to the lungs and multiple other organs, occasionally causing death. Until effective vaccines are developed to curb the pandemic, it is paramount to define the mechanisms and develop protective therapies to prevent organ dysfunction in patients with COVID-19. Individuals that develop severe manifestations have signs of dysregulated innate and adaptive immune responses. Emerging evidence implicates neutrophils and the disbalance between neutrophil extracellular trap (NET) formation and degradation plays a central role in the pathophysiology of inflammation, coagulopathy, organ damage, and immunothrombosis that characterize severe cases of COVID-19. Here, we discuss the evidence supporting a role for NETs in COVID-19 manifestations and present putative mechanisms, by which NETs promote tissue injury and immunothrombosis. We present therapeutic strategies, which have been successful in the treatment of immunο-inflammatory disorders and which target dysregulated NET formation or degradation, as potential approaches that may benefit patients with severe COVID-19.

## Facts

- Patients with COVID-19 show signs of dysregulated innate and adaptive immune responses.
- SARS-CoV-2-induces the formation of NETs through ACE2 and requires active TMPRSS2 and virus replication.
- Immunothrombosis triggered by NETs mediates damage of distant organs.

## Open questions

- Would inhibition of neutrophil proteases ameliorate tissue injury in patients with COVID-19?
- How are neutrophils and NETs influenced by a network of antibodies, complement proteins, clotting factors, CRP, nucleases, proteases, and anti-proteases?
- Does the modulation of NET formation and its clearance complement current therapies?
- Can the synergism of DNases and heparin in NET degradation be exploited as co-adjuvant therapy?

## Basic aspects of neutrophil biology and their relevance for COVID-19

Neutrophils normally differentiate in the bone marrow and throughout this process start to express effector molecules that are stored in granules allowing them to mount inflammation and kill microbes [1, 2]. A distinctive feature of mature neutrophils is that they cannot proliferate and, thus stay for only short periods in the circulation [3]. Mature neutrophils transit from the bone marrow into the circulation and from the circulation into the tissues even under steady-state conditions. The trafficking and reactivity of neutrophils to pathogens follow circadian patterns [4–6]. The influx of neutrophils from the circulation into tissues happens in most organs, but in particular in highly vascularized ones such as lungs and kidneys, representing the prime targets in coronavirus disease 19 (COVID-19) [7]. Notably, although neutrophils homing into tissues have partially lost their prestored molecules [5], they remain active and can damage vessels and parenchyma. The neutrophil to lymphocyte ratio has been identified as the most important independent risk factor for severe COVID-19 [8].

Mechanisms underlying neutrophil development have received increasing attention [9–13]. There is still no unified nomenclature for neutrophil developmental stages, which would be particularly useful to understand emerging observations in the context of COVID-19 and other disorders. For simplicity, we adopt here a naming system recently proposed [14], in which neutrophil development transits from proNeu1 via proNeu2, preNeu, and immature to mature neutrophils in the bone marrow. Immunophenotyping of COVID-19 blood samples revealed that the emergence of immature subsets of neutrophils (preNeu and immature) in the blood correlates with severe COVID-19, suggesting that precise delineation of neutrophil subsets could be used as a predictive marker for COVID-19 severity [15–17].

## Mechanisms of neutrophil extracellular trap formation

Neutrophils are prompted to release neutrophil extracellular traps (NETs) upon encounter of danger signals (Supplementary Fig. 1), which in essence are structures composed of DNA decorated with histones and granule proteins such as lactoferrin, cathepsins, neutrophil elastase (NE), and myeloperoxidase (MPO) (Supplementary Fig. 2), as well as cytoplasmic and cytoskeletal proteins [18, 19]. Mitochondrial DNA is also found in NETs [20, 21]. NETs immobilize pathogens, limit their dissemination, and enable their killing by antimicrobial proteins. Beyond antimicrobial defense, there is growing evidence that NETs contribute to the pathogenesis of numerous diseases due to either excessive formation and/or impaired removal, which turns out to be toxic for the host [22].

**Fig. 1:**
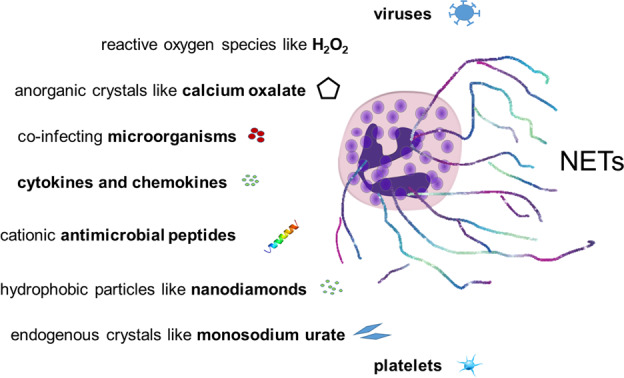
Potential mediators for the induction of NET-formation in the infected and inflamed tissues. Viruses (SARS-CoV-2), ROS, calcium oxalate, co-infecting microorganisms, cytokines and chemokines, cationic antimicrobial peptides, nanodiamonds, monosodium urate (MSU), and platelets reportedly induce NET formation. See main text for references. Original illustration from the authors.

Activation of neutrophils through Toll-like receptors, G protein-coupled receptors, Fc-, chemokine- and cytokine- receptors can stimulate NET formation (Fig. 1). Neutrophil activation by engagement of these receptors induces NET formation by various mechanisms, many of which are linked to the activation of the NADPH oxidase (NOX) complex. However, NOX-independent processes have also been described to lead to the NET formation [23]. Reactive oxygen species (ROS) produced in the context of NOX activation and mitochondrial dysfunction [21] are important in the rearrangement of the cytoskeleton [24] and glycolytic ATP production [25], which are required for NET formation. Early during NET formation, granular NE and MPO translocate to the nucleus and drive nuclear and chromatin decondensation [26]. Peptidylarginine deiminase 4 (PADI4) contributes to chromatin decondensation by histone hypercitrullination [27]. Citrullination licenses calpain to further decondensed nuclei before extracellular trap release [28]. However, like for NOX, PADI4- and NE-independent pathways have also been reported [29, 30]. In addition, necroptotic and pyroptotic pathways can be activated [31]. Cathepsin C (CatC) also plays an important role since it is required for the activation of NE and other serine proteases [32].

**Fig. 2:**
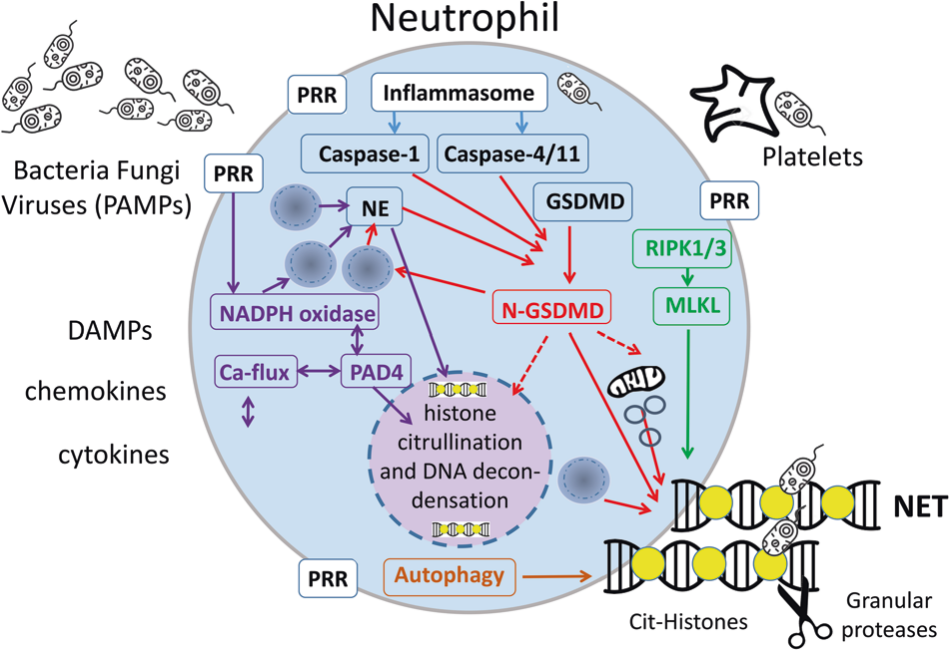
Mechanisms of NET formation. Pathways that regulate NET formation (see body text for references). Pattern recognizing receptors (PRR) initiate NADPH oxidase activation and a spike of cytosolic calcium activating neutrophil peptidylarginine deiminase 4 (PADI4) causing histone citrullination (yellow circle) and DNA decondensation. Chromatin and/or mitochondrial DNA is expelled and form NETs. Several necrotic cell death pathways may contribute to NETosis. Necroptosis involves RIPK1/RIPK3-mediated activation of MLKL and plasma membrane permeabilization contributing to the release of NETs. Pyroptosis involves canonical or non-canonical inflammasome activation by the caspases-1 or 4, respectively. Caspase-1 and 4 as well as NE cleave GSDMD and generates the N-GSDMD fragment with a pore-forming activity that enables the release of NETs. In addition, autophagic processes contribute to the release of NETs. Original illustration from the authors.

During NET formation, NE also cleaves gasdermin D (GSDMD), a molecule centrally involved in pyroptosis, and constituting a feed-forward loop to facilitate granule and plasma membrane permeabilization (Fig. 2) [33]. Conversely, NET formation is facilitated after cytosolic LPS sensing and caspase-11-dependent activation of GSDMD [34]. Disulfiram interferes with the papain-like proteases of the SARS-COVID-19 infection cycle [35] and was shown to modify Cys^191^–Cys^192^ in GSDMD to reduce pore [36] and possibly NET release.

## Pro- and anti-inflammatory functions of NET

**Fig. 3:**
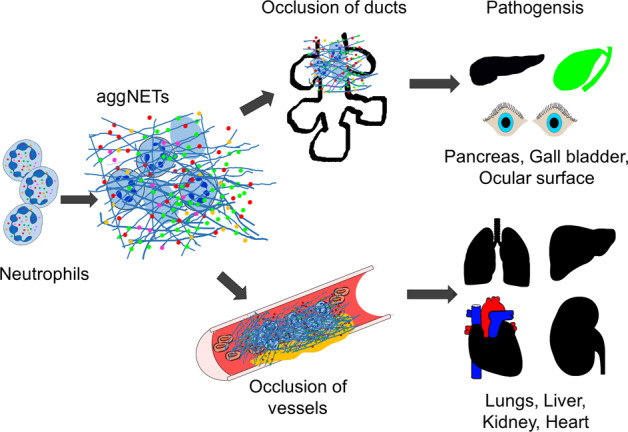
The role of aggregation and degradation of NETs in vascular occlusions. Increased numbers of patrolling neutrophils in inflamed tissues form aggregated neutrophil extracellular traps (aggNETs). These are prone to occlude the ducts and glands of the pancreas, gall bladder, and ocular surface. The occlusions precipitate organ pathogeneses like pancreatitis and cholelithiasis. AggNETs also occlude blood vessels in particular the microvasculature of lungs, liver, kidney, heart, and thus cause pathogenesis. Original illustration from the authors.

In general, NETs can exert both pro- and anti-inflammatory effects, which are context-dependent [37]. Proinflammatory effects include the induction of type I Interferons (IFNs) [38] and proinflammatory cytokines [21], induction of the NLRP3 inflammasome [39], promotion of adaptive immune responses [40], damage to the endothelium [41], and immunothrombosis [42]. In addition, NET aggregation can occlude ducts in various organs and promote organ damage [43, 44]. Indeed, pre-clinical strategies to interfere with the release of NETs or to promote the clearance of formed NETs can prevent organ injury in numerous models of inflammatory diseases [45, 46]. On the flip side of the coin, aggregation of NETs can promote the trapping and cleavage of proinflammatory mediators by NET-bound proteases (Fig. 3) [47–49], eventually leading to downregulation of inflammatory responses and resolution of inflammation.

## NETs activation

### Viral infections induce the formation of NET

**Fig. 4:**
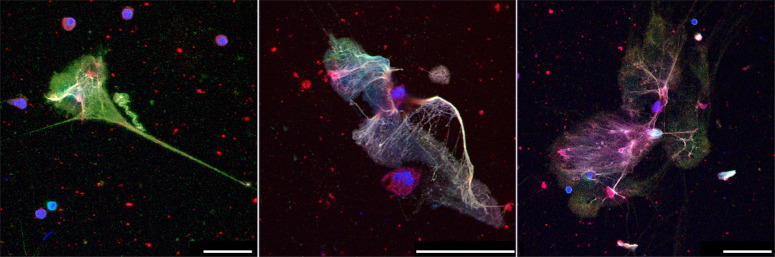
NETs induced by SARS-CoV-2. NET formation of human blood-derived neutrophils after treatment with SARS-CoV-2. Immunofluorescence staining of NETs was done using antibodies against elastase (red) and DNA-histone1-complexes (green), with a counterstain of DNA (blue). Yellow staining indicates colocalization of NETs (histone-DNA fibers) with elastase. The Bars represent 25 µm (left) and 50 µm (middle and right). Original illustration from the authors.

A wide array of pathogens triggers NET formation [18]. These include viruses such as a respiratory syncytial virus (RSV) [50] and influenza [51]. Initial studies showed that sera from COVID-19 patients triggered NET release by healthy control neutrophils in vitro [52] and more recent evidence suggests that viable SARS-CoV-2 can directly stimulate human neutrophils to release NETs in a dose-dependent manner (Fig. 4) [53]. SARS-CoV-2-mediated NET-induction requires the angiotensin converting enzyme 2 receptor (ACE2), expressed by neutrophils, the activity of the serine protease TMPRSS2, and virus replication. Similar to what was observed for RSV, the pan-PAD inhibitor Cl-Amidine abrogated SARS-CoV-2 induced NET formation, implying that inhibition of NET formation may represent a potential therapeutic option for COVID-19.

### The role of citrullination in NETs

The physiological NET formation is typically associated with PADI4 activation (Fig. 2) [54]. PADI4 converts positively charged arginines to neutral citrullines in protein substrates, including core histones [55]. Citrullination unleashes the energy of coiled DNA, leading to the catapult-like ejection of NETs [56]. PADI4 retains enzymatic activity in the extracellular environment and modifies proteins, including those of the extracellular matrix [57] and coagulation factors [58]. Accumulation of citrullinated histones was found in COVID-19 and in influenza-infected mice [52, 59, 60]. Since pan-PADI and PADI4 inhibitors such as Cl-amidine, BB-CL-amidine, YW-56, or GSK484 have shown efficacy in the treatment of NET-mediated pathologies, such as lethal lung endotoxemia [61] and cellular damage due to hypoxia [62], the administration of such inhibitors may prove beneficial for the treatment of COVID-19.

### The relation of platelet activation with NETs

Platelets are activated during COVID-19, forming aggregates with leukocytes, in particular in patients with severe disease [59, 63–67]. Platelets are well known to adhere to injured blood vessels, become activated, and express adhesion molecules, including P-selectin and ICAM-1, leading to neutrophil recruitment. Platelets—due to their number and privileged position in the blood—may represent major instigators of neutrophil activation [68] through direct contact [69]. The physiological importance of this interaction may be to trigger neutrophil-mediated repair [70, 71]. Circulating platelets do not spontaneously bind neutrophils, but do so in the context of bacterial [72] or viral infection [73]. This interaction relies on integrins [74] and may result in NET formation. Indeed, platelets can trigger NET formation (Fig. 2), but the platelet-derived molecules that induce NET release remain poorly characterized. While a consensus is lacking, HMGB1 [75] and inorganic polyphosphate (polyP) [76] are candidates to underlie this phenomenon. However, this mechanism is debated [77]. Thus, platelet activation may trigger the formation of intravascular NET aggregates in the pulmonary and renal microcirculation [59, 65], thereby contributing to the manifestations of COVID-19 [78].

### Complement activation as a trigger for NETs

Complement activation fosters the cytokine storm and coagulopathy, both critical events in COVID-19 [79]. A history of macula degeneration, associated with complement-activation, predisposes to poor outcomes during COVID-19, while complement deficiencies appear to be protective [79]. SARS-CoV-2 activates compliment and complement regulators [79] and consistently C5a and C5b-9 accumulate in the blood of COVID-19 patients, indicating complement activation [80, 81]. Complement deposition is detected in the microvasculature, occasionally in proximity to SARS-CoV-2 glycoproteins [82]. Complement activation may thus represent an additional trigger for NETs also in COVID-19 [81].

## NETs’ impact at the cellular level

### The role of NET-bound enzymes

Neutrophil granules contain various serine proteases including NE, cathepsin G, and proteinase-3, lactoferrin, MPO, and lysozyme that can promote tissue damage [45]. These enzymes, which also appear in NETs, can modulate viral immune responses through modification of autoantigens and immune complexes [83]. NE can further cleave the spike protein and thus activate the fusogenic peptide of SARS-CoV-2 spike protein S2 [84]. These findings suggest that the proteolytic activity of neutrophil-derived enzymes may modulate membrane fusion of the virus [85]. The effects of NE may be modulated by protease inhibitors such as serum alpha-1-antitrypsin (serpinA1) to prevent tissue injury and virus activation. Increased serum NE activity was detected during severe COVID-19, despite the functional inhibitory activity of serpinA1 against exogenous soluble NE [59], thereby revealing a mechanism of resistance of NET-derived NE to serpinA1 that may be relevant during COVID-19 [86].

### NET-induced thromboinflammation in COVID-19

Thrombotic complications contribute to morbidity and mortality in severe COVID-19 [87, 88]. Thrombosis in patients with COVID-19 affects both the arterial and venous circulation, leading to acute coronary syndrome, stroke, deep vein thrombosis, pulmonary embolism, and microvascular thrombosis (Fig. 3) [89–92]. The NET-remnants, including circulating cell-free DNA, citrullinated H3, or MPO-DNA complexes, are abundantly found in the circulation of patients with severe COVID-19 [52, 93]. Furthermore, neutrophil-platelet aggregates and neutrophil activation markers are also increased in patients with severe disease [65, 94]. Importantly, NETs from patients with COVID-19 are loaded with tissue factor (TF). Complement activation has been linked to the release of thrombogenic NETs decorated with TF [81]. The excessive NET formation may also cause direct vascular injury [41, 95] and indirectly support the formation of autoantibodies that determine the appearance of various forms of autoimmune vasculitis [96, 97]. Along this line, histopathology studies have shown that NET-based immunothrombosis is linked to organ damage in severe COVID-19 [98]. Lung autopsies from patients with COVID-19-related acute respiratory distress syndrome (ARDS) revealed widespread occlusion of small pulmonary vessels by aggregated NETs [93]. Neutrophils also infiltrate alveolar and interstitial areas of K18-hACE2 transgenic mice infected with SARS-CoV-2 leading to a comparable pulmonary pathology [32, 99]. NET-rich thrombi, platelets, and fibrin were also present in the lung, heart, and kidney [59, 65]. This clogging of microvessels by aggregated NETs (aggNETs) may contribute to fatal outcomes in COVID-19. Notably, in steady-state conditions, DNAses prevent vascular occlusions by non-canonical NET-driven thrombosis [100]. This observation indicates that NET-dissolving mediators can also be impaired or overwhelmed in the patients [101].

### NET-induced endothelial activation and damage in COVID-19

Endothelial injury is considered an essential pathogenic process in COVID-19, leading to lung and kidney damage [102–104]. Organ- and microenvironment-associated endothelial heterogeneity likely contributes to different COVID-19 outcomes [105]. Similar to other SARS viruses, SARS-CoV-2 enters cells through ACE2, expressed on renal and pulmonary endothelial cells [106, 107]. In accordance, SARS-CoV-2 has been detected intracellularly in renal and pulmonary endothelial cells [107, 108]. Paracrine factors released from infected endothelial cells [109] may impact disease outcome by altering functions of epithelial or other neighboring cells, including neutrophils and pneumocytes. Furthermore, endothelial damage fosters perivascular T-cell recruitment and disrupts the alveolar-capillary barrier in the lungs [91]. Acute endothelial damage in COVID-19 is associated with structurally deformed capillaries and signs of compensatory neovascularization [91]. This compromised endothelial barrier triggers lung edema and proteinuria, which are common observations in severe lung and kidney diseases [91, 110, 111].

NETs directly activate endothelial cells, induce endothelial to mesenchymal transition, and apoptotic endothelial cell death. Thus, NETs compromise endothelial integrity and barrier function and promote endothelial dysfunction (Supplementary Fig. 3) [41, 112, 113]. Since NETs are abundant in the circulation and in lung and kidney tissues of patients with COVID-19 [65], their accumulation represents a key trigger to induce pulmonary and renal microvascular thrombosis, which triggers disease-related organ failure [59, 81, 93, 114]. The effect of classical anti-thrombotic treatments may be hampered as NETs have shown to be central components of vascular occlusion in COVID-19 [100].

## NETs’ impact at the organ level

### NETs in COVID-19-associated acute lung disease

**Fig. 5:**
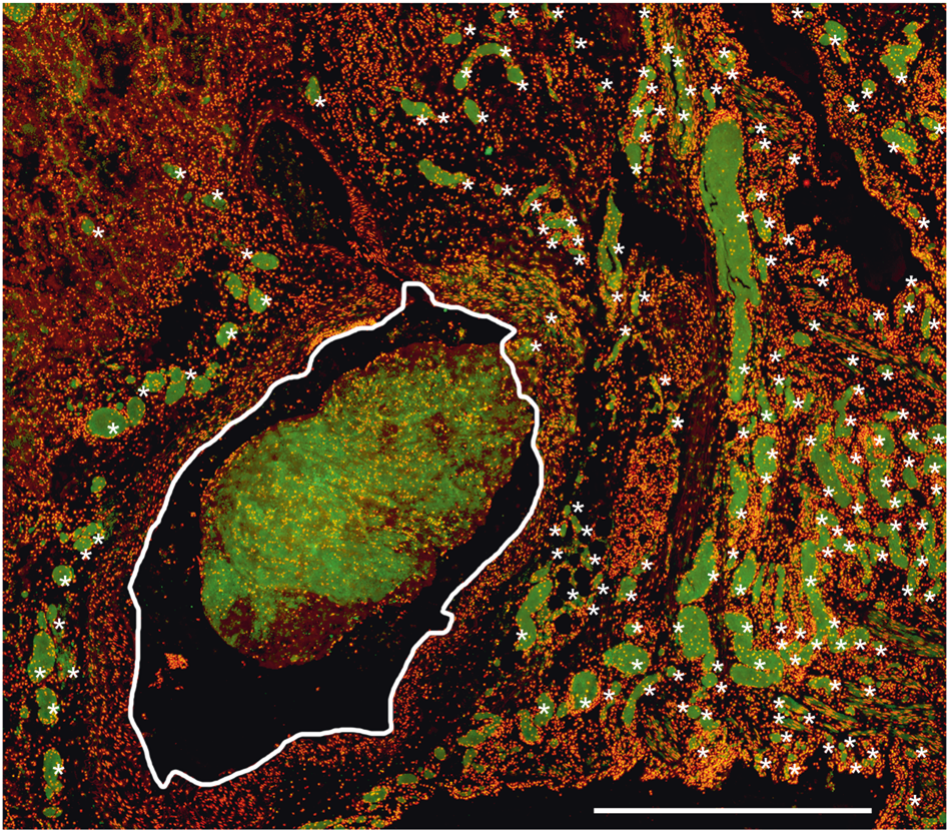
Occlusion of pulmonary vessels by aggNETs in COVID-19. Occlusion of small and intermediate-sized pulmonary vessels in COVID-19 published by Leppkes et al 2020 [59]. The former is marked by asterisks and the latter by a white frame. Note, that large fields of the (micro)-vasculature are occluded by NETs identified by extracellular neutrophil elastase (green). The nuclei of the cells were stained with propidium iodide (red). The bar represents 1000 µm. Original illustration from the authors.

Histopathological studies revealed that respiratory symptoms and shortness of breath in COVID-19 occur secondary to alveolar-capillary damage, hemorrhage, immune cell infiltration, fibrin deposition, and fluid-filled alveoli [115–118]. Detailed analysis of lungs revealed abnormal extracellular matrix remodeling, denuded alveolar epithelia, and proliferation of epithelial cells and fibroblasts. Importantly, neutrophilia directly correlates with disease severity in COVID-19 [102]. Increased serum levels of neutrophil-derived MPO-DNA and citrullinated histone H3, both NET degradation products, closely parallel lung distress and predict COVID-19 severity [52]. Furthermore, circulating nucleosomes were identified as potential markers to monitor COVID-19 disease progression [119]. Immature and low-density neutrophils predominate in severe COVID-19 [15, 16, 120]. Neutrophils that recently emigrate from the bone marrow have higher granule contents and enhanced NET release, which aggravates pulmonary injury in murine models [5]. It is therefore conceivable that immature neutrophils in the circulation of COVID-19 patients actively promote susceptibility to ARDS [5]. Likewise, hypogranular neutrophils produced during emergency myelopoiesis have a higher propensity to release NETs and may be causally related to COVID-19 severity [93]. NETs released by SARS-CoV-2–activated neutrophils promote lung epithelial cell death in vitro [53, 121]. In this line, COVID-19 goes along with massive infiltration of neutrophils into the lungs, including the formation of NETs as potential drivers of ARDS [122] and the associated immunothrombosis of patients with COVID-19 (Fig. 5) [59, 93].

### NETs in kidneys and liver

NET formation contributes to numerous forms of acute and subacute kidney injury with proteinuria [123]. NOX-independent NETs directly induce kidney endothelial dysfunction, thereby offering a potential explanation for the proteinuria observed in most patients with COVID-19 [112]. NET-rich microvascular thrombi were also detected in the autopsy material of the kidneys in severe COVID-19 with renal failure [65]. It is still elusive whether involvement relates to the renal tropism of SARS-CoV-2 or to a systemic tendency for immunothrombosis and cytokine storm (Supplementary Fig. 4) [124, 125].

Liver injury emerges as a co-existing symptom in COVID-19 [126] and it might result from direct viral toxicity, but also from an overproduction of cytokines and/or NETs [127]. In patients with COVID-19 injured liver displays patchy necrosis alike in experimental models of the net-damaged liver [128, 129].

## Targeting NETs in COVID-19 treatment

### Glucocorticoids, hydroxychloroquine, and heparin in NET formation

COVID-19 patients frequently receive dexamethasone [130], heparin [131], and until recently also hydroxychloroquine, the latter emerged as highly controversial and not beneficial for the course of the disease [132–134]. The anti-inflammatory action of hydroxychloroquine relies on the inhibition of lysosomal activity and cytokine production. In vitro, neutrophils are more prone to release NETs when exposed to chloroquine [135]. The effect of hydroxychloroquine administered in vivo regarding NET formation has not been systematically studied yet [136–138]. In contrast, glucocorticoids including dexamethasone have been reported to reduce NET formation [139] most likely by suppressing the expression of inflammatory mediators that activate neutrophils. As mentioned above, activated neutrophils and platelets play key roles in thrombosis associated with severe COVID-19 [65]. Excessive NET formation harbors the risk of vascular occlusion [59], while heparin reduced NET formation in an experimental in vivo model of lung injury [140]. Heparin and low molecular weight heparins neutralize extracellular cytotoxic histones [141, 142], accelerate DNaseI-mediated degradation of NET mediated clots [59], and prevent NET aggregation by nano- and microparticles [143] in COVID-19. The therapeutic value of heparin in COVID-19 has been demonstrated recently [144], though some patients may develop heparin resistance [145].

### Cytokine inhibitors and NET formation

Given the reduced incidence of COVID-19 in individuals treated with cytokine inhibitors [146] and the promising results with IL-6 and IL-1-blockade and immunosuppressants [130], inflammatory cytokines are likely important players in mediating inflammatory tissue damage in response to SARS-CoV-2. Stimulating results have been obtained with tocilizumab in a randomized clinical, double-blind, placebo-controlled, phase III study (preprint) [147] dampening the late IL-6-driven hyper-inflammatory phase [148] and reducing the need for mechanical and non-invasive ventilation. However, mortality after 28 days was not affected in another randomized fully peer-reviewed clinical trial [149]. The IL-1 receptor antagonist Anakinra is also currently under evaluation in RCTs, following early encouraging results in observational studies [150, 151]. Clinical studies with JAK-STAT inhibitors, also inhibiting IL-6, in addition, also IFNs, are still ongoing (NCT04320277). JAK inhibitors and direct blockade of IL-6 inhibit NET formation [152]. Previous studies in murine models have shown that the JAK inhibitor tofacitinib impairs NET formation in vitro and in vivo [153].

### Complement-based therapies

Thrombogenic NETs elicited by the activated complement present in the blood of patients with COVID-19 [81] are candidates to amplify inflammation and thrombosis [154, 155]. Anti-complement strategies based on eculizumab or AMY-101 have successfully been used in small numbers of severe and critical/intubated patients [156–160]. Eculizumab is an anti-C5 humanized monoclonal antibody clinically approved for selected rare complement-mediated disorders. AMY-101 is clinically developed for various complement-mediated disorders and belongs to compstatin, a group of small-sized peptides that bind C3 and prevent its activation [160]. Early clinical data indicates that both inhibitors resulted in the resolution of SARS-CoV-2-associated ARDS; however, AMY-101 was associated with a more robust reduction in circulating neutrophils and NETs, highlighting the role of C3 in NET-driven thromboinflammation [160]. Several compassionate use programs or Phase II RCTs with complement inhibitors are in progress (NCT04346797, NCT04355494, NCT04288713, NCT04395456, EudraCT2020-004408-32).

Together with the complement components C1q-C4, C-reactive protein (CRP) functions in the disposal of bacteria and apoptotic or necrotic host cells [161, 162]. As COVID-19 is characterized by high CRP levels, it was proposed that reduction of the CRP levels by therapeutic apheresis, might reduce the pathological process in early disease [163]. Agarose bead-based CRP adsorption from the blood additionally depleted cell-free chromatin co-aggregates with C3 fragments [164]. The role of this approach in COVID-19 remains to be determined. In addition to CRP, calprotectin was identified as another acute-phase protein in severe pulmonary disease in COVID-19 [165].

### Modulation of purinergic signaling

Injured cells release ATP that signals “danger” to neighboring tissues [166]. As a counterpoint, ectonucleotidases hydrolyze ATP to generate adenosine that supports local homeostasis. Activation of specific surface adenosine receptors suppresses NET formation via cyclic AMP-dependent signaling [167, 168]. Dipyridamole is an inexpensive, FDA-approved drug with a favorable safety profile. Dipyridamole potentiates adenosine receptor signaling by (i) inhibition of ectonucleoside reuptake, and (ii) stabilization of intracellular cyclic AMP. Dipyridamole tempers NET release in vitro while preventing NET-dependent thrombosis in mice [168]. In a small study, dipyridamole suppressed D-dimer levels in patients with COVID-19 [169]. Larger studies are required to evaluate clinical outcomes (NCT04391179) [170].

### Treatment with DNases

Recombinant DNAse1 efficiently breaks down the chromatin of NETs that contributes to immunothrombosis and luminal obstructions of airways and vessels [59, 171]. NET-driven mucus accumulation, rigidity, and airway occlusion in severe COVID-19 may benefit from the same treatment [172, 173]. A small single-center case series (preprint) suggested that nebulized endotracheal DNAse1 (Dornase) reduced supplemental oxygen requirements in the patients [174]. COVIDornase (NCT04355364) and COVASE (NCT04359654) are two current initiatives that evaluated nebulized dornase α in prospective randomized controlled multicentre trials [175]. Since DNase1L3, which degrades extracellular DNA, works in a tandem with DNase1 to prevent immunothrombosis in an animal model of leukophilia [100], they are both candidates for the treatment of vascular occlusions in COVID-19. However, it is important to highlight that digestion of extracellular DNA with DNase1 and/or DNase1L3, while potentially reducing the occlusive capacity of aggregated NETs, may not successfully remove remnants that retain pro-inflammatory activities (Fig. 2) [176].

### Other interventions to inhibit the NET formation

Treatment options targeting the pro-inflammatory action of NETs such as PADI4 inhibitors, or antibodies that block extrusion of NETs [46], or R406, a potent SYK inhibitor and the metabolically active component of fostamatinib [177], are potential new classes of drugs to tackle NET formation and to alleviate NET toxicity and in patients with severe COVID-19 [178]. In addition, the pharmacological inhibition of CatC to counterbalance the unwanted effects of neutrophil serine proteases in severe COVID-19 is considered a potential therapeutic target [179]. Some of these mediators are already in the development pipelines of pharmaceutical companies awaiting clinical trials. After the successful implementation of glucocorticoids [130], and the positive data on routine heparin use [180], future therapies will have to show how they perform compared to this standard of care. The studies of tocilizumab in severe COVID had heterogeneous outcomes [181, 182]. The patient cohort that had the most benefits from tocilizumab was mostly cotreated with glucocorticoids. This points to possible combination therapy as a future strategy. Combination therapy may also be useful for NET-targeted therapies: blocking new NET formation and improving degradation of preformed NETs. Given that NET degradation is negatively affected in severe COVID, the synergistic effect of DNase1 and heparin in NET degradation [59] may have identified these agents as suitable combination partners to effectively improve NET degradation in severe COVID. This needs to be proven by future clinical studies.

## Conclusion

Here, we have highlighted the multifaceted functions that NETs play in the pathogenesis of COVID-19. The role of NETs in COVID-19 is increasingly supported by multiple lines of evidence and in fact, explains the wide range of manifestations seen in the most severe and critical cases. In the pathophysiology of COVID-19, there appears to be an important role for neutrophil dysregulation, oxidative stress, and aberrant NET formation as well as clearance. Neutrophils and NETs are at the crossroads of innate immune responses like pathogen killing, thrombosis, and activation of the adaptive immune system. This cardinal position helps to understand why a dysregulated neutrophil response upon SARS-COV-2 infection leads to such severe and uncontrolled disease manifestations. The pleiotropic complications caused by deposition of NETs in vessels and tissues in fact match disease manifestations in patients with COVID-19, and demand urgent actions to set trials with NET inhibitors. Identifying subgroups of individuals at risk for neutrophil dysregulation following SARS-CoV-2 exposure may help further refine individualized therapies. This strategy aims to prevent devastating complications including lung injury, kidney damage, endotheliitis, and immunothrombosis in severe COVID-19.

### Search strategy and selection criteria

The data for this review were identified through searches of MEDLINE, PubMed, and references from relevant articles using the search terms “neutrophils” and “COVID-19” or “immunothrombosis” and “neutrophils”. Abstracts and reports from meetings were excluded. It uses 683 articles published in English from 2014 to April 2020. The publication date of additional articles was unlimited.

## Supplementary information

Supplementary figures
