# Supplementary material for: Patients with COVID-19: in the dark-NETs of neutrophils
Source: Cell Death Differ. 2021 May 24;28(11):3125–39. doi: 10.1038/s41418-021-00805-z (PMC8142290; doi:10.1038/s41418-021-00805-z)
Supplement: Supplementary file 1 — Supplementary figures [file 41418_2021_805_MOESM1_ESM.pdf]

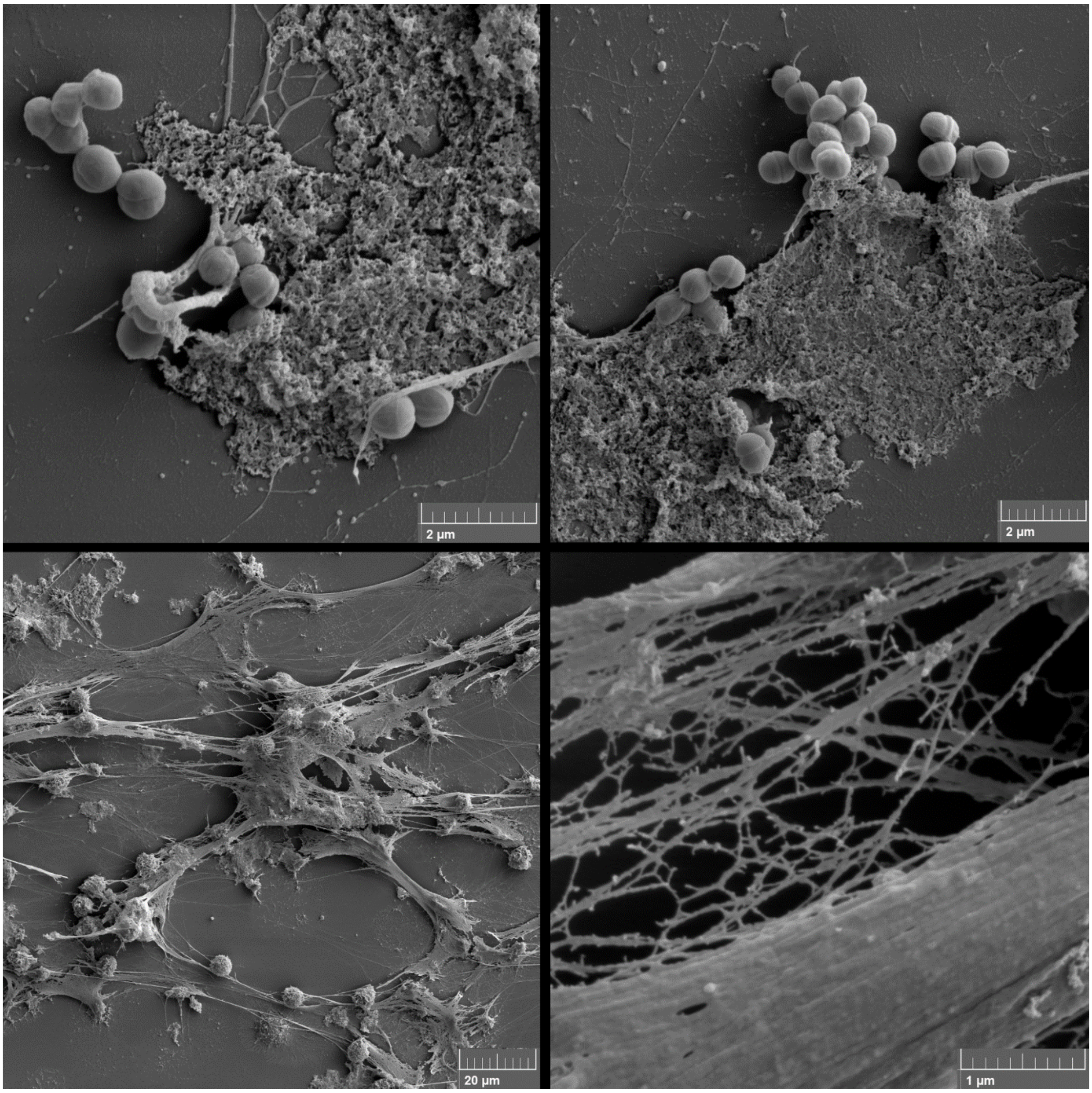

Supplementary figure 1. Display of neutrophil extracellular traps (NETs) in scanning electron microscopy (SEM).

The upper panel shows bacteria trapped in NETs. Lower panel shows neutrophils incubated with LPS from *Klebsiella pneumoniae*. Higher resolution showed the fine structure of NETs as fibrous DNA bundles. Original illustration from the authors.

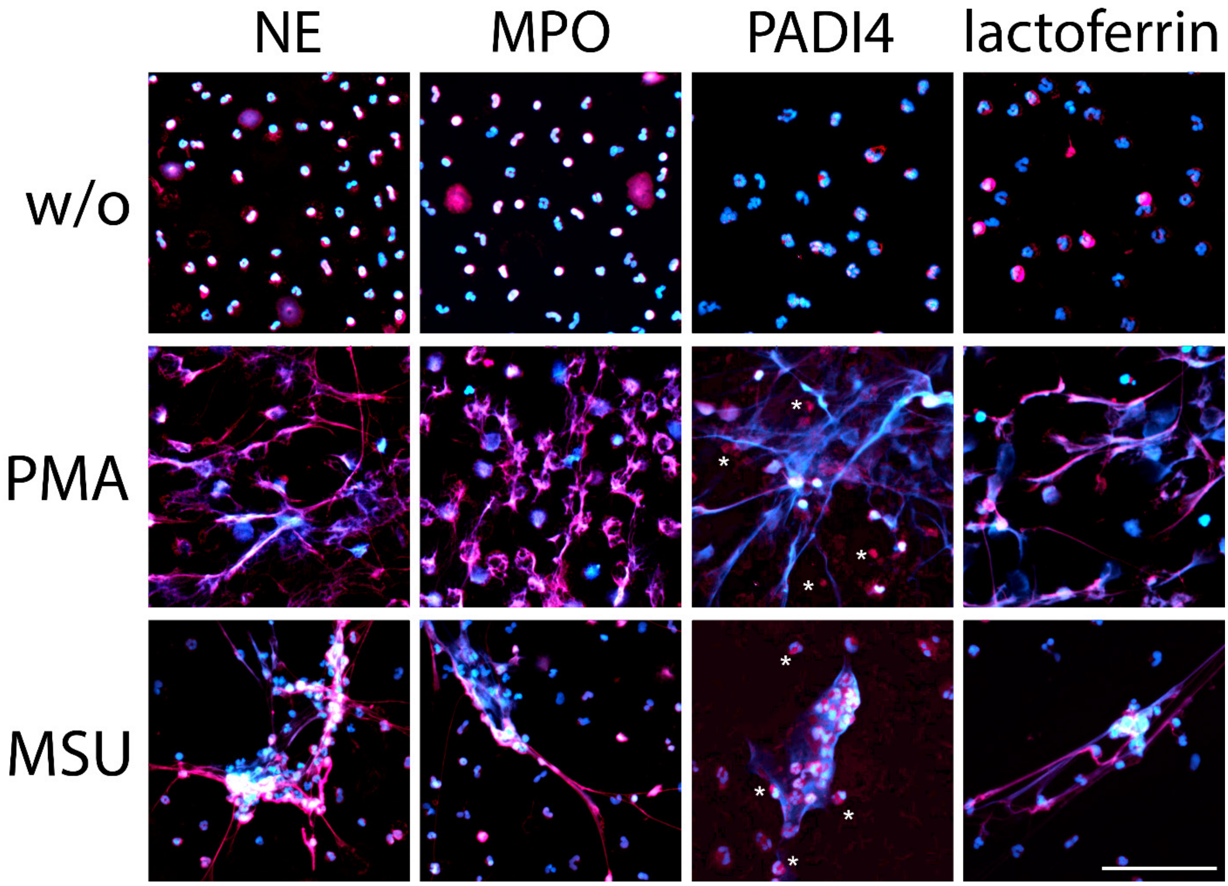

Supplementary figure 2. NET-associated proteins.

Human neutrophils were treated for 4 h with Phorbol-12-myristat-13-acetat (PMA) or monosodium iodide (MSU) or left untreated. Then, the cells and NETs were stained for neutrophil elastase (NE), myeloperoxidase (MPO), Peptidyl Arginine Deiminase-4 (PADI4), and lactoferrin (all displayed in red). Note, that the granular proteins NE, MPO, and lactoferrin remain associated with the NETs, whereas most of the cytoplasmic and nuclear PADI4 is released as large complex (marked with asterisks). Viable cells with lobulated nuclei are not stained for these intracellular antigens. Hoechst 33342 (displayed in blue) served as counterstain for DNA. The bar represents 100 µm. Original illustration from the authors.

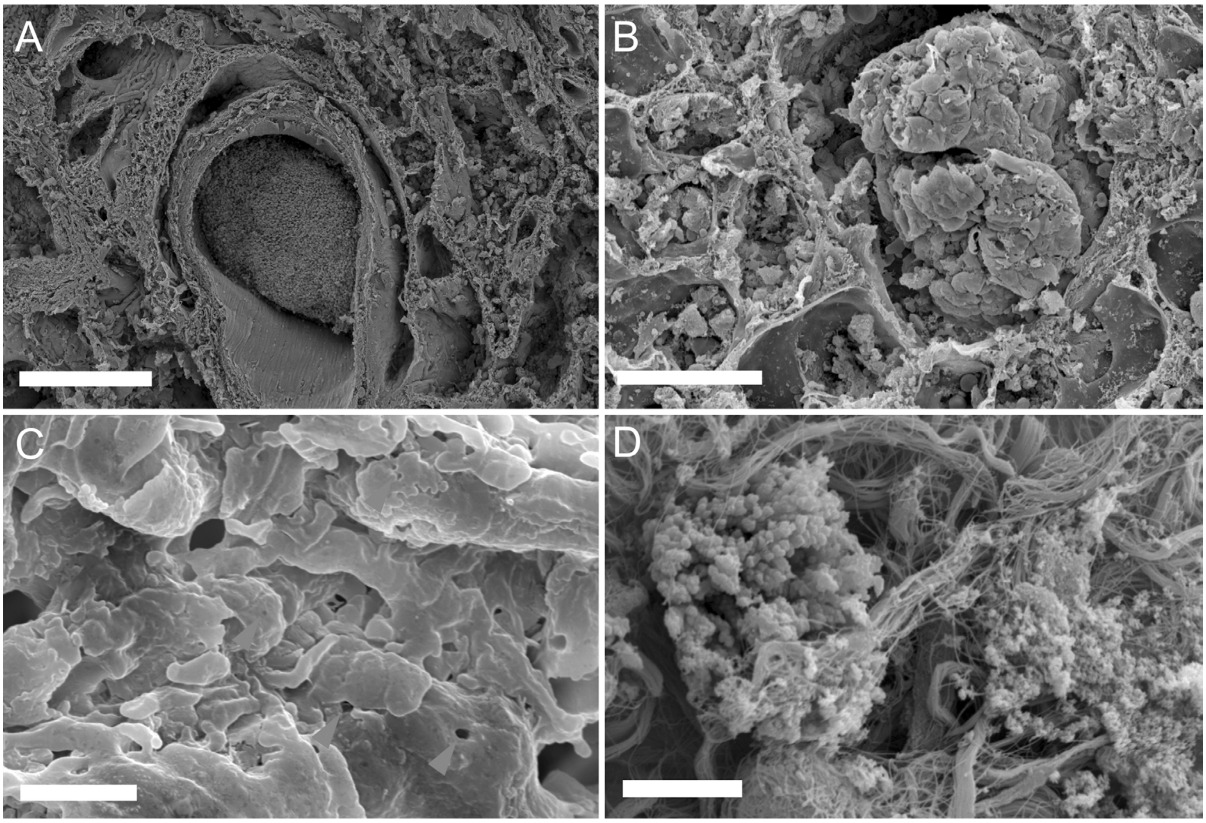

Supplementary figure 3. Endothelial dysfunction in COVID-19.

Endothelial dysfunction related to SARS-CoV-2 infection is predominantly associated with (A) pulmonary edema, microthrombi and (B) glomerular injury; (C) the endothelial dysfunction causes a unique healing reaction called intussusceptive angiogenesis by splitting of existing blood vessels to form new ones; (D) microthrombi are associated with the formation of NETs. The bar represents 100µm (A), 20µm (B), 10µm (C) and 2µm (D). Original illustration from the authors

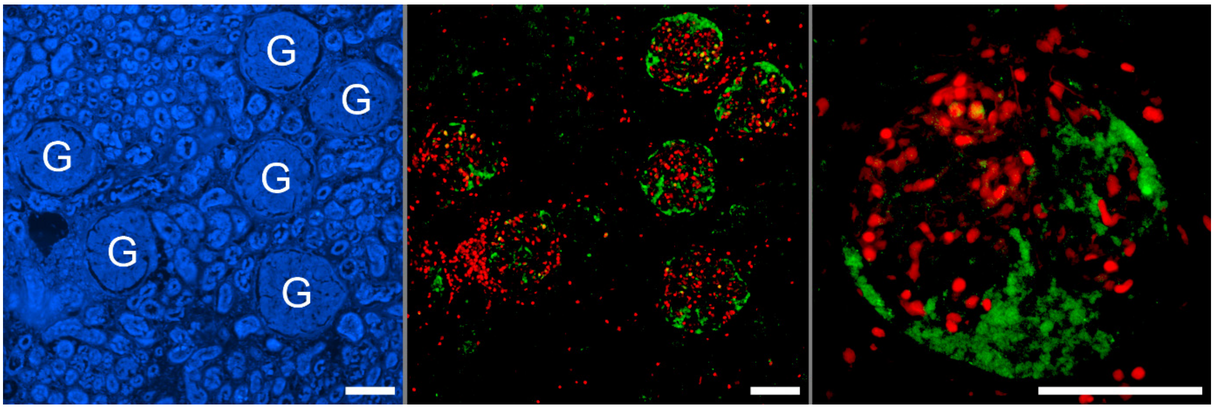

Supplementary figure 4. Occlusion of renal glomeruli by aggNETs.

Endothelial damage in the glomeruli of a patient with COVID-19 causes accumulation in the Bowman's space of aggNETs. Left: Natural fluorescence of renal autopsy material is shown. Glomeruli are marked with "G". Middle: neutrophil elastase (green) clotting the Bowman's space. Right: Glomerulus in higher magnification. PI stained nuclei are shown in red. The size bars represent 50 µm. Original illustration from the authors.
